# The Efficacy of Laser Doppler Flowmetry, Electric Pulp Test and Cold Test in Diagnosing Revascularization of Extrusively Luxated Immature Maxillary Incisors

**DOI:** 10.12669/pjms.344.15524

**Published:** 2018

**Authors:** Seyda Ersahan, Fidan Alakus Sabuncuoglu, Elif Aybala Oktay

**Affiliations:** 1 Seyda Ersahan, Department of Endodontics, Faculty of Dentistry, Istanbul Medipol University, Istanbul, Turkey; 2 Fidan Alakus Sabuncuoglu, Department of Orthodontics, Faculty of Dentistry, Health Sciences University, Ankara, Turkey; 3 Elif Aybala Oktay, Department of Restorative Dentistry, Faculty of Dentistry, Health Sciences University, Ankara, Turkey

**Keywords:** Dental trauma, Electric pulp testing, Extrusion injury, Immature teeth, Laser Doppler Flowmetry, Pulpal blood flow, Thermal testing

## Abstract

**Objective:**

To evaluate the effect of extrusion on immature permanent tooth PBF values during a 6-month post-trauma period and to compare the accuracy of cold tests on pulp sensibility of traumatized teeth with that of electric pulp tests (EPT).

**Methods:**

The study group comprised of 26 extruded immature maxillary incisors in 25 trauma patients. The respective contralateral homologous teeth (*n*=25) were included as a positive control group. Teeth in the study group were treated by repositioning and splinting. Pulp vitality readings for traumatized and control teeth were taken with LDF, EPT and cold test on the day of splint-removal (2-3 weeks after trauma–T1) as well as 6 weeks (T2), 3 months (T3) and 6 months (T4) post-trauma. Student *t* and Mann-Whitney *U*-tests were used to compare data among groups. Statistical significance was set at *P*< 0.05.

**Results:**

LDF gave positive vitality readings (>4.5 PU) in all patients from T1 to T4 (with the exception of 1 patient at T1).

**Conclusions:**

LDF was able to accurately identify vitality in traumatized immature teeth even during the first few weeks following trauma, whereas conventional sensibility tests were unable to accurately recognize vitality shortly after trauma.

## INTRODUCTION

Luxation injuries of permanent teeth are true dental emergencies that occur most frequently in children between the ages of 8 and 15.^1^ One of the most serious of these injuries is extrusive luxation, in which the tooth is centrally displaced from its socket and exhibits increased mobility. Extrusive luxation is a complex wound involving disruption of the periodontal ligament, cementum and pulp neurovascular supply.^2^

Pulp necrosis has been reported to be the most common complication after extrusive injuries.^2^ However, correctly diagnosing necrosis is particularly challenging for the pediatric dentist, particularly in young children and in cases of dentoalveolar trauma. Pulp vitality is commonly measured using thermal and electrical sensibility tests^3^, which evaluate pulp vitality indirectly by measuring pulpal nerve response, rather than directly monitoring vascularity. However, teeth that have temporarily or permanently lost sensory function due to trauma may not respond to sensibility testing despite the presence of an intact vasculature.^4^

Direct measurement of pulpal blood flow (PBF) provides the most accurate and reliable assessment of pulp status.^5^ Laser Doppler flowmetry (LDF) is a well-documented, non-invasive technique that provides direct, objective measurements of blood circulation. Flowmetric values have been used to accurately identify the reestablishment of vitality in traumatized teeth.^6^-^8^ Data on PBF values in children with traumatized immature permanent maxillary incisors is scarce, with two traumatized immature teeth in a single patient.^9^ Therefore, the current study aimed to evaluate the effect of extrusion on immature permanent tooth PBF values during a 6-month post-trauma period and to compare the accuracy of cold tests on pulp sensibility of traumatized teeth with that of electric pulp tests (EPT).

## METHODS

The study was conducted with one study group and two control groups. The study group (*n* = 26) was comprised of traumatically extruded immature permanent maxillary incisors identified during clinical and radiographic examinations of patients applying to the hospital for treatment. Inclusion criteria were as follows:

1. Extrusive luxation
2. Immature roots and open apices
3. Presence of a contralateral homologous tooth with no signs/symptoms of injury
4. Recentness of trauma (< 24 h)

Exclusion criteria were as follows:

1. Complete root formation
2. Patient history of caries, restorations, or repeated dental trauma
3. Crown and/or root fracture.

In total, 26 teeth in 26 patients met these criteria and were included in the study group (mean age: 8.3 years). A positive control group (*n* = 26) was comprised of contralateral teeth, and a negative control group (*n*=8) was comprised of endodontically treated mature maxillary incisors. The study design was approved by the hospital ethics committee (No: 37732058-53), and informed consent was obtained from all participants and their parents.

### Repositioning and splinting

Prior to treatment, baseline periapical radiographs were obtained to confirm the diagnosis of extrusion. Pulp sensibility tests were also performed initially. Each extruded incisor was gently repositioned in its alveolus and stabilized using a passive, non-rigid splint. In line with current guidelines for semi-rigid fixation, the splints were kept in place for a minimum of 14 days and a maximum 21 days.^10^

### Recording sessions

Pulpal conditions were evaluated at six time points. Teeth in the study group and the positive control group were measured on the day that the splint was removed from the traumatized tooth (2-3 weeks after extrusion – T1) and 6 weeks (T2), 3 months (T3) and six months (T4) after trauma. Measurements of teeth in the negative control group were performed at similar time intervals. In order to assess and compare the different methods, all three tests were performed on all teeth in the same order (EPT, cold, LDF), with 30 minutes between each test.

### Electrical test

Electric pulp testing was performed on all teeth using a Parkell pulp sensibility tester (Parkell Electronics Division, Farmingdale, New York, USA). The tooth to be tested was isolated and the probe was placed on the labial surface of the tooth. The intensity of electric current was slowly increased by turning a dial on the tester from 0 to 10 at a rate of one numerical increase per five seconds until a response was obtained.^3^ The positive control tooth was tested first, followed by the contralateral traumatized tooth. The average number at which a response was elicited was calculated and recorded. If no response was elicited from the patient even at maximum intensity, then the test tooth was considered nonvital.^3^ Negative control teeth were subjected to EPT testing using the same procedures.

### Thermal (cold test)

A thermal (cold) test was performed on all teeth by soaking a cotton pellet with 1,1,1,2 Tetrafluoroethane (-26.2°C, Endo ice refrigerant spray; Coltene / Whaledent Inc., Mahwah, New Jersey, USA) and placing it on the middle third of the facial surface of each tooth to be tested for 15 seconds. The test was repeated after a 2-minute interval, and a tooth was rated as having no response to cold if the patient felt no sensation with either application.

### Laser Doppler flowmetry

PBF measurements were performed with a laser Doppler flowmeter (Periflux PF 4001; Perimed, Jarfalla, Sweden). Light with a wavelength of 632.8 nm was produced by a 1 mW He–Ne laser within the flowmeter and transmitted along a flexible fiber-optic conductor (125 µm dia.) inside a specially designed 2-mm-dia. round dental probe (PF 416; Perimed), and the backscattered light was returned from the tooth to the flowmeter along a second fiber (fiber to-fiber distance: 500 µm). The amount of Doppler-shifted light returned is processed by the flowmeter, which produces an output signal with a voltage that is linearly related to the flux of red blood cells (number of cells x average velocity) encountered within the tooth and represents a relative measure of PBF. The flowmeter was calibrated prior to each data collection session.

**Fig.1:**
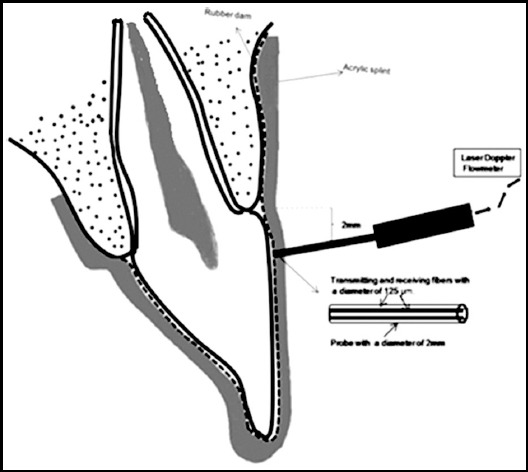
Diagram of the experimental set up.

In order to obtain accurate, reproducible measurements, a custom-fabricated, self-curing acrylic-resin splint was used to secure the probe in the appropriate position approximately 3 mm from the gingival margin (Fig.1). PBF of the experimental and control incisors was measured during the same session. Prior to measurement, a rubber-dam and splint were positioned in the patient’s mouth. For each measurement session, the mean PU for each tooth was calculated based on the phase of stable values, excluding peaks attributable to movement artefacts. LDF data were transferred to a computer connected to the RS-232 port of the flowmeter using the system’s own software (PeriSoft for Windows, Perimed) and stored for later analysis.

### Statistical analysis

Statistical analysis was performed using the MedCalc Statistical Software, version 12.7.7 (MedCalc Software bvba, Ostend, Belgium; http://www.medcalc.org; 2013). Student *t-*test was used to compare data between normally distributed groups, and the Mann-Whitney *U*-test was used to compare data between non-normally distributed groups, with non-parametric statistical methods used for values with skewed distribution. Changes in measurement values between time points were evaluated by paired sample *t*-test for normally distributed data and by Wilcoxon Signed Rank test for non-normally distributed data. A χ² test was used to evaluate positive and negative responses to sensibility tests. EPT and Cold Test results were compared using a McNemar test and Kappa statistics. A two-sided *P* value of <0.05 was considered statistically significant.

## RESULTS

Mean PBF values are summarized in Table-I. Mean LDF readings for teeth in the study group increased steadily from T1 (4.8 ± 1.2 PU) to T3 (7.7 ± 1.3 PU), reaching the same levels as the positive controls at T3 (*P*=0.938). PBF values in the study group were significantly lower than in the positive controls at T1 (*P*<0.001) and T2 (*P*=0.026), whereas no significant differences were found between mean PBF values of the two groups at T3 (*P*=0.938) or T4 (*P*=0.831). Paired sample *t* and Wilcoxon signed rank tests showed no significant changes in PBF in either the positive or negative control groups over the course of the study; however, PBF values of the study group varied significantly over time (except T3-to-T4: *P*=0.341).

**Table-I:** LDF recordings of PBF in all groups.

		T1		T2		T3		T4	

	n	mean±sd	Med(min-max)	mean±sd	Med (min-max)	mean±sd	Med (min-max)	mean±sd	Med (min-max)
Study group	25	4.8±1.2	4.3 (3.4-6.9)	6.6±1.6	6.6(4.5-9.6)	7.7±1.3	7.8(5.4-9.7)	7.7±1.3	7.8(5.4-9.8)
Positive control	25	7.7±1.3	7.8(5.4-9.7)	7.7±1.3	7.8(5.4-9.8)	7.7±1.3	7.8(5.4-9.7)	7.7±1.3	7.8(5.5-9.8)
Negative control	8	0.7±0.5	0.7(0.1-1.4)	0.8±0.5	0.7(0.2-1.5)	0.7±0.4	0.7(0.1-1.4)	0.7±0.5	0.6(0.1-1.5)
P^1^		<0.001*, <0.001**		0.026*, <0.001**		0.938*, <0.001**		0.831*, <0.001**	

1 Mann-Whitney U test,

* Comparison between study and positive control group,

** Comparison between study and negative control group

EPT and cold test results are summarized in Table-II and Table-III, respectively. A McNemar test showed no differences between EPT and cold test results for the positive control group (*P*=0.250). *P*-values could not be calculated for either the study group or the negative control group using a McNemar test (Table-IV); however, a Kappa test found EPT and cold test results for the study group to be in agreement at T2 (*К*=0.627), T3 (*К*=0.918) and T4 (*К*=0.752) and at all times for the positive control group (T1: *К*=0.516; T2: *К*=0.651; T3: *К*=0.519; T4: *К*=0.519).

**Table-II:** Response to EPT in all groups.

N(%)		T1		T2		T3		T4	
	
	n	(-)	(+)	(-)	(+)	(-)	(+)	(-)	(+)
Study group	25	25 (100)	0	23 (92)	2 (8)	11 (44)	14 (56)	6 (24)	19 (76)
Positive control	25	5(20)	20 (80)	6 (24)	19 (76)	7 (28)	18 (72)	7 (28)	18 (72)
Negative control	8	8(100)	0	7 (87.5)	1 (12.5)	6 (75)	2 (25)	6 (75)	2 (25)
P^1^		<0.001*,		<0.001*		0.024*		1.00^2^*	

1 Chi-Square test,

* Comparison between study and positive control group

**Table-III:** Response to cold test in all groups.

		T1		T2		T3		T4	

	n	mean±sd	Med (min-max)	mean±sd	Med (min-max)	mean±sd	Med (min-max)	mean±sd	Med
Study group	25	24 (96)	1 (4)	21 (84)	4 (16)	10 (40)	15 (60)	4 (16)	21 (84)
Positive control	25	2 (8)	23 (92)	5(20)	20 (80)	3 (12)	22 (88)	3 (12)	22 (88)
Negative control	8	0.7±0.5	0.7(0.1-1.4)	0.8±0.5	0.7(0.2-1.5)	0.7±0.4	0.7(0.1-1.4)	0.7±0.5	0.6(0.1-1.5)
P^1^		<0.001*,<0.001**,		0.026*, <0.001**		0.938*, <0.001**		0.831*, <0.001**	

1 Chi-Square test,

^2^Fisher Exact test,

* Comparison between study and positive control group.

**Table-IV:** Inter-rater agreement between EPT and cold test.

N (%)	Cold (-)	Cold (+)	Total
*Study Group*			
EPT (-)	24 (96)	1 (4)	25 (100)
EPT (+)	0	0	0

*Positive Control*			

EPT (-)	2(40)	3(60)	5(100)
EPT (+)	0	20(100)	20(100)

*Negative Control*			

EPT (-)	8(100)	0	8(100)
EPT (+)	0	0	0

A comparison of PBF values and responses to sensibility tests is given in Table-V. In both the study and positive control groups, a negative response to sensibility testing was associated with lower PBF values when compared to teeth that showed a positive response.

**Table-V:** Association between the PBF values and pulp response to sensibility tests.

			T1 mean±SD (n)		T2 mean±SD (n)		T3 mean±SD (n)		T4 mean±SD (n)	

		N	(-)	(+)	(-)	(+)	(-)	(+)	(-)	(+)
EPT	Study group	25	4.8±1.2 (25)	-	6.4±1.5 (23)	9.4±0.4 (2)	6.4±0.8 (11)	8.6±0.7 (14)	5.9±0.3 (6)	8.2±1.0 (19)
	Positive control	25	5.8±0.3 (5)	8.1±1.0 (20)	5.9±0.4 (6)	8.2 ±1.0 (19)	6.4±1.3 (7)	8.2±1.0 (18)	6.2±0.8 (7)	8.2±1.0 (18)
	Negative control	8	0.7±0.5 (8)	-	0.8±0.5 (8)	-	0.8±0.5 (7)	0.5 (1)	0.7±0.5 (7)	0.6 (1)
Cold	Study group	25	4.9±1.2 (24)	3.6 (1)	6.2±1.4 (21)	8.9±0.7 (4)	6.4±0.8 (10)	8.5±0.8 (15)	5.8±0.3 (4)	8.0±1.1 (21)
	Positive control	25	5.8±0.5 (2)	7.8±1.2 (23)	5.9±0.4 (5)	8.1 ±1.1 (20)	5.9±0.3 (3)	7.9±1.2 (22)	5.9±0.3 (3)	7.9±1.2 (22)
	Negative control	8	0.7±0.5 (8)	-	0.7±0.4 (7)	1.5 (1)	0.6±0.4 (6)	1.1±0.4 (2)	0.7±0.5 (6)	0.8±0.7 (2)

## DISCUSSION

This study evaluated pulp vitality in traumatically extruded immature permanent incisors using LDF and compared the results with conventional methods for testing pulp sensibility (EPT and cold tests) over a 6-month post-trauma period. This is the first study to investigate the effect of extrusive injuries on PBF changes in immature permanent teeth. More than 90% of traumatized teeth failed to respond to either sensibility test at T1 (2-3 weeks) and T2 (6 weeks), whereas more than 75% of contralateral control teeth responded positively to both sensibility tests at T1 and T2. The lack of response to sensibility tests at T1 and T2 could be a result of trauma-related injury, inflammation, pressure, or tension in nerve fibers in the apical area.^11^ Recovery was observed to have begun at T3 (3 months), with more than half of the traumatized teeth that had not responded to initial tests showing a positive response at T3. By T4 (6 months), the rate of positive response to sensibility tests among traumatized teeth had increased to a rate similar to that of non-traumatized controls (>75%). This corroborates the theory that the transition from a negative to a positive response to sensibility tests may be explained by the transient nature of the damage to pulpal nerve fibers^12^ and reinforces the fact that in many cases, nerve paresthesia in traumatized teeth resolves itself over time and that normal responsiveness will eventually be regained following injury.^6^

LDF data in this study showed the mean PBF in traumatized teeth to be significantly lower than that of the contralateral control teeth when measured after splint-removal 2-3 weeks post-trauma (T1). The fact that LDF measurements of both control groups showed no statistically significant changes in mean PBF values during the study period indicates the initial reduction in PBF registered in traumatized teeth to be unrelated to repeated measurement, flowmeter calibration, or test sensitivity. Rather, the low PBF values at T1 may be attributed to either the damaging effect of traumatic force on the neurovascular bundle entering the apical canal opening^11^ or to the effect of the splint applied as treatment. In the current study, the vascular disturbances observed in the traumatized teeth gradually improved after T1, so that at three months post-trauma (T3), there was no longer any significant difference in the PBF values of traumatized and control teeth. The recovery in PBF at T3 may be an indication that the pulpal circulatory system of immature teeth has the capacity to compensate for reductions in pulpal blood flow. Whereas LDF registered signs of vitality (>4 PU)^13^ in all teeth (100%) even at T1, EPT and cold tests registered vitality in only 76% and 84% of traumatized teeth, respectively, at T4. These findings support the assumption that neural regeneration in traumatized teeth is slower than vascular regeneration and in some cases may not occur at all.^12^-^14^ It also highlights the advantage of using LDF rather than traditional sensibility tests to monitor incisors during the immediate post-trauma phase.

The finding of significant reductions in PBF during the first few weeks post-trauma is in line with many earlier studies.^6^,^8^,^15^-^17^ Moreover, some of these studies^8^,^17^ reported a correlation between LDF measurements and the severity of adverse trauma-related outcomes, with PBF levels of < 3 PU significantly associated with pulp necrosis, periapical radiolucency and crown discoloration. In the present study, PBF levels never dipped below 3 PU in any of the traumatized teeth, and even at T1, the mean PBF value for traumatized teeth was 4.8 PU. Only one study in the literature^7^ reported LDF to show similar levels of vitality in traumatized and control teeth. In that study, no significant differences were found in LDF signal levels between controls and 16 out of 20 traumatized teeth (unresponsive to EPT); however, given that the study population included only subluxation injuries, which are less severe than extrusive injuries, these findings are not particularly surprising.^7^

The present study’s finding that PBF gradually recovered to a point where there were no longer any significant differences in PBF values of traumatized teeth and controls is in line with a previous LDF study by Gazelius B et al.^6^ That study reported PBF of subluxated teeth to be arrested at one week, partially restored at six weeks and to regain normal values at 9 months post-trauma. The faster recovery in our study (3 months post-trauma) could be due to an improved capacity of incompletely developed teeth with open apical foramina to tolerate some degree of trauma-related displacement of the root apex. In contrast to the findings of the present study, an LDF study by Strobl H et al.^16^ found the PBF of mature teeth with extrusive luxations to remain significantly lower than control teeth throughout the course of follow-up (from splint-removal to 36 weeks following splint removal) and to show no significant improvements over time, a possible indication of pulpal necrosis. Despite the number of previous clinical studies concluding that luxation injuries can have a permanent, iatrogenic effect on pulpal circulation^15^-^17^, it is difficult to correlate past findings with those of the present study due to differences in injury type (a variety of injury subtypes in previous studies vs. extrusive luxation only in this study) and tooth maturity (mature incisors in previous studies vs. immature incisors in this study).

The present study’s finding that pulp often fails to respond to sensibility tests during the initial period following traumatic injury, but that this lack of response is temporary and is usually resolved in 6 months, is supported by numerous previous studies.^2^,^3^,^6^,^11^ For example, Andreasen JO et al.^11^ and Skieller V et al.^18^ reported that up to one-half of all luxation injuries will show no response to sensibility testing immediately after trauma, and Gopikrishna V et al.^3^ found that positive responses to thermal/electric pulp tests increased from 0% on the day of trauma to 94% after three months.

A number of previous studies have compared the accuracy of LDF and sensibility tests.^19^,^20^ Mesaros SV et al.^19^ assessed the vitality of avulsed maxillary central incisors in one patient using both LDF and a cold test. While one of the incisors remained weakly responsive and the other was still unresponsive to CO_2_ ice even at 76 days after replantation, LDF indicated revascularization to be occurring in both teeth at a much earlier time (3 weeks).^19^ Similarly, in a study by Roeykens H, et al.^20^ 2 severely luxated central incisors involved in a clinically and radiographically detectable alveolar bone fracture were unresponsive to cold tests, whereas reproducible values indicating vitality were obtained for LDF starting at 7 weeks. These findings are in line with the findings of the present study.^19^,^20^ While the present study found similar reliability for EPT and cold tests in detecting vitality in immature traumatized teeth (Mc Nemar test, *P*=0.250), LDF was found to be more effective in detecting vitality than both EPT and cold tests, with 20% of teeth that did not respond positively to sensibility tests shown to possess vitality by LDF (at T4). Furthermore, negative responses to sensibility tests in both traumatized and positive control teeth were associated with lower PBF values. These findings highlight the advantage of using LDF rather than traditional sensibility tests to make a true evaluation of pulpal vitality of traumatized immature teeth at an early stage. LDF usage may thus be suitable to reach an early decision on whether treatment like apexification and regenerative endodontic procedures after luxation injuries is needed.

Because of the immediate need to provide emergency, LDF could not be performed when the patient presented with trauma. While understandable, this lack of data represents an important methodological limitation of this study. Even if it were possible to construct a splint to provide accurate repositioning at this time, the pain and anxiety experienced during trauma, along with gingival hemorrhaging and the use of a local anesthetic solution during repositioning could influence PBF measurements taken during the first few hours following trauma, and the splint used for tooth repositioning could itself affect PBF measurements. In order to eliminate the effects of all these variables, the first LDF measurements were delayed until the day of splint removal.

This study demonstrated LDF to be an effective, objective method of evaluating pulp vitality in recently traumatized immature incisors and one that is capable of recognizing the potential for healing at a relatively early stage. Consistent LDF readings in this study confirmed that pulp circulation can be detected by LDF even in the first few weeks following trauma. No difference was found in the reliability of thermal and electric pulp tests for identifying sensibility of traumatized immature teeth; however, when compared to LDF, neither of these methods recognized vitality until much later than LDF, and a relatively large proportion of teeth (20%) remained unresponsive to sensibility tests even at six months post-trauma.

### Author’s Contribution

**SE** conceived, designed and did statistical analysis & editing of manuscript.

**FAS & SE** did data collection and manuscript writing.

**EAO** did review and final approval of manuscript.
